# Design of a Robust Synchronization-Based Topology Observer for Complex Delayed Networks with Fixed and Adaptive Coupling Strength

**DOI:** 10.3390/e26060525

**Published:** 2024-06-18

**Authors:** Yanqin Sun, Huaiyu Wu, Zhihuan Chen, Yang Chen, Xiujuan Zheng

**Affiliations:** 1 School of Information Science and Engineering, Wuhan University of Science and Technology, Wuhan 430081, China; sunyq923@163.com (Y.S.);; 2 School of Mechatronics and Intelligent Manufacturing, Huanggang Normal University, Huanggang 438000, China; 3 Institute of Robotics and Intelligent Systems, Wuhan University of Science and Technology, Wuhan 430081, China

**Keywords:** complex delayed networks, topology identification, robust-synchronization-based observer, fixed and adaptive coupling strength

## Abstract

Network topology plays a key role in determining the characteristics and dynamical behaviors of a network. But in practice, network topology is sometimes hidden or uncertain ahead of time because of network complexity. In this paper, a robust-synchronization-based topology observer (STO) is proposed and applied to solve the problem of identifying the topology of complex delayed networks (TICDNs). In comparison to the existing literature, the proposed STO does not require any prior knowledge about the range of topological parameters and does not have strict limits on topology type. Furthermore, the proposed STO is suitable not only for networks with fixed coupling strength, but also for networks with adaptive coupling strength. Finally, a few comparison examples for TICDNs are used to verify the feasibility and efficiency of the proposed STO, and the results show that the proposed STO outperforms the other methods.

## 1. Introduction

As we all know, complex networks have become ubiquitous as models of various real-world systems. In order to analyze the statistical properties and behaviors of networks, researchers have extensively studied the topology and dynamics of complex networks [1,2]. Ref. [1] explored the statistical mechanics of network topology and dynamics, while ref [2] analyzed the impact of time-delay coupling on the synchrony of gene networks composed of coupled oscillators. Furthermore, numerous case studies have been conducted on different real-world networks, yielding insights from various perspectives. These studies have encompassed predicting collective behaviors [3,4], applications in control systems [5,6], and other areas. These achievements have made significant contributions to the theory of multi-connected models, which significantly increases the working efficiency of networked systems. But it is worth noting that much complex network research is based on the assumption that the network topology is known or predefined. However, with the high security and secrecy requirements of complex networks in practice, due to the strict industrial system protection mechanism, some parameters are difficult to measure. Therefore, we focus primarily on identifying the topology of complex networks in this paper.

Generally speaking, in the most complex networks, time lag is unavoidable due to the time lag characteristics of certain physical devices and real-world networks. In order to simulate more realistic networks, there has been pioneering research in the topology identification of complex delayed networks (TICDNs). Some initial results have been obtained. A number of the existing approaches, such as optimization-based strategies [7,8,9,10,11,12], compressed sensing technologies [13,14,15,16], and synchronization-based methods [17,18,19,20,21,22,23,24,25], are widely used to deal with unknown topology. The main idea of an optimization-based strategy is to convert the TICDN into an optimization problem and then solve the problem with various classical or evolutionary algorithms. In [8], a chaotic ant swarm intelligence algorithm (CASIA) was investigated to identify topology. Other classical algorithms, such as weighted least square (WLS) algorithms, for the identification of the topology of uncertain networks were studied in [10]. The unknown topological structure of power systems was studied in [11], which was formulated as a weighted least square problem and solved by a distributed sub-gradient algorithm (DSA). On the other hand, compressed sensing technology uses a special sampling mechanism to recover the original signal for the TICDN, which has the advantage of requiring less measurement data. In [13,14], the researchers devoted their efforts to study the topology recovery of single and multiple complex networks based on compressive sensing and Taylor expansion. In [15], the authors proposed a method combining compressive sensing and graph theory to identify smart grids.

However, while optimization-based strategies accurately estimate unknown topology, their computational costs are often high. Compressed sensing does not apply to some non-sparse networks, and the sparse representation of the signals must be incoherent, which is not straightforward to achieve in real networks. Along with the development of modern control theorems, the TICDN problem based on synchronization methods has gradually attracted the attention of researchers in recent years, This method has been explained in detail and proven rigorously via the Lyapunov stability theory [26], Linear Matrix Inequalities (LMI) [27], and many other techniques [28,29]. Unknown topology can be identified via the proposed STO, and the response–drive networks have to actualize synchronization. In the literature mentioned above, topology observers are designed for complex networks via the sliding mode control technique [18], the adaptive-feedback approach [19,20,21,22,23,24], and the line feedback approach [25]. With further research, considering the large scale of some networks, pinning control has played a certain role because only a small number of nodes require control. In [30], the authors addressed the synchronization problem of linear coupled complex networks via aperiodically intermittent pinning control. The authors of [31] mainly studied the synchronization of neural networks via the pinning control of certain vital nodes. The authors of [32] studied the pinning synchronization problem for a class of complex networks using a moment-based analysis approach. Recently, in order to save on the cost of identification and investigate the benefits of the pinning mechanism, the authors of [33,34] investigated the problem of identifying the partial topology for undirected networks.

Compared to the optimization strategy and compressed sensing-based technology, the synchronization-based method avoids the need for both huge computations and a priori knowledge about topological parameters. Meanwhile, unknown topology is not necessarily required to be symmetric, irreducible, or sparse. Owing to these features, more unknown network models have been further investigated via the synchronization-based method [35,36,37]. On the other hand, it should be noted that the majority of the above-mentioned studies focus on the coupling strength being a known constant coefficient in network models. This may sometimes be unsatisfactory, as some real-world networks are characterized by evolution [38]. That is to say, the coupling strength is adaptively tuned based on the environment and human activities in various cases. In real life, there are some examples of complex networks with adaptive coupling strength. For instance, the phenomenon of stochastic resonance in networks with small-world connectivity has been investigated when the coupling strength is adaptive [39]. Swarms or flock of robots can adjust the frequency of information exchange itself under varying environmental and working conditions [40]. That is, the strong coupling relationship between multiple agents becomes a weak coupling relationship at another time. A second-order Kuramoto model with adaptive coupling strength was investigated in [41]. It was found that adaptive coupling strength is more flexible than fixed coupling strength for the activation of synchronization in complex networks. Comparatively speaking, complex networks with adaptive coupling strengths naturally emerge and cannot be overlooked, which motivated the current work.

To the best of our knowledge, there are few results regarding research on TICDNs with adaptive coupling strength. Inspired by previous discussion, we present a systematic investigation into the online estimation of the uncertain topology in complex delayed networks. The main contributions of this paper are as follows:

(1) A parameter observer-based approach named the synchronization-based topology observer (STO) method is proposed in this study. The STO mainly includes a series of ingenious controllers and topology observers. Different from traditional adaptive control schemes without the effects of input delays, we propose a hybrid non-delayed and delayed output feedback control scheme. Additionally, the adaptive laws of the proposed controllers ${\dot{k}}_{i}(t)=f(k_{i}(t),e_{i}(t))$ instead of classic traditional adaptive laws ${\dot{k}}_{i}(t)=f(e_{i}(t))$ can help to shorten the system’s adjustment and organization time.

(2) A newly designed update law of coupling strength is presented. Adaptive coupling strength is considered in a complex delayed-network model during the identification process, which can be automatically tuned to the optimal value. According to Lyapunov stability theory, a strict theoretical proof is given.

(3) The relevant comparison results show that the proposed STO has higher identification ability and better robustness to the variations in parameter and external disturbance than the other methods.

The remainder of this paper is organized as follows. In Section 2, the model description and parameter identification approach are briefly outlined. The main theory for identifying the topology of complex networks is given in Section 3. In Section 4, some numerical simulations and comparisons are chosen to validate the effectiveness of the proposed method. Finally, our conclusions are summarized in Section 5.

## 2. Problem Formulation

### 2.1. Model Description

In this paper, we considered a general continuous-time complex delayed network including N identical nodes, in which the ith node is described as

(1) $${\dot{x}}_{i}(t)=f(x_{i}(t))+c(t)\sum_{j=1}^{N}a_{ij}\Gamma x_{j}(t-\tau )\;\;i=1,2\cdots ,N$$

where $x_{i}(t)={\left[x_{i1}(t),\;x_{i2}(t),\cdots ,x_{in}(t)\right]}^{T}\in R^{n}$ is the state vector; f(⋅) is a nonlinear function; $A=\left(a_{ij}\right)\in R^{N\times N}$ is the outer coupling matrix (defined as follows: If there is a connection between i*th* node and jth node, then $a_{ij}\neq 0\;(i\neq j)$; otherwise, $a_{ij}=0$(i≠j)); the diagonal elements with indices i=j are defined as $a_{ii}=-\sum_{i=1,i\neq j}^{N}a_{ij}$ (i=1,2……N); c(t) is the coupling strength between nodes; τ is the coupling delay; and $\Gamma \in R^{n\times n}$ is the inner coupling matrix. Before giving the main result, some useful mathematical preliminaries are given.

**Lemma** **1** (See [22])**.** *For any vector* $x,y\in R^{n}$ *and a positive definite matrix* Q*, the following inequality holds:* $2x^{T}y\leq x^{T}Qx+y^{T}Q^{-1}y$.

**Assumption** **1.** *Topological matrix* $A=(a_{ij}{)}_{N\times N}$ *satisfies* $\left|a_{ij}\right|\leq l$*, where* l *is a bounded positive constant.*

### 2.2. Topology Identification Based on Synchronization

In this section, the method based on synchronization for the TICDN is introduced. Model (1) is assigned as the drive complex network with a state variable $x_{i}(i=1,2,\ldots ,N)$, the response complex delayed network is described as follows:

(2) $${\dot{y}}_{i}(t)=f(y_{i}(t))+c(t)\sum_{j=1}^{N}{\hat{a}}_{ij}\Gamma y_{j}(t-\tau )+u_{i}(t)\;\;i=1,2\cdots ,N$$

where $y_{i}(t)={\left[y_{i1}(t),\;y_{i2}(t),\cdots ,y_{in}(t)\right]}^{T}\in R^{n}$ is the response state vector of the i-*th* node. $\hat{A}=\left[{\hat{a}}_{ij}\right]\in R^{N\times N}$, and $u_{i}(t)$ is the i-*th* controller that needs to be designed.

**Definition** **1.** *The drive network (1) and the response network (2) are considered to have achieved complete synchronization if the synchronization error* $e_{i}(t)$ *is satisfied as follows:*

(3) $$\lim_{t\to \infty }\left\|e_{i}(t)\right\|=\lim_{t\to \infty }\left\|y_{i}(t)-x_{i}(t)\right\|=0\;\;\;i=1,2,\ldots ,N$$

**Definition** **2.** *The topology* $a_{ij}$ *in network (1) is said to be identified if*

$$\lim_{t\to \infty }({\hat{a}}_{ij}(t)-a_{ij})=0\;\;i,j=1,2,\ldots ,N$$

From the networks in Equations (1) and (2), the synchronization error between the drive and response networks is described as follows:

(4) $$\begin{array}{cc} {\dot{e}}_{i}(t) & ={\dot{y}}_{i}(t)-{\dot{x}}_{i}(t)\\ & =f(y_{i}(t))+c(t)\sum_{j=1}^{N}{\hat{a}}_{ij}\Gamma y_{j}(t-\tau )+u_{i}(t)-\left\{f(x_{i}(t))\right.+c(t)\sum_{j=1}^{N}a_{ij}\left.\Gamma x_{j}(t-\tau )\right\}\\ & =f(y_{i}(t))-f(x_{i}(t))+c(t)\sum_{j=1}^{N}({\hat{a}}_{ij}-a_{ij})\Gamma y_{j}(t-\tau )+c(t)\sum_{j=1}^{N}a_{ij}\Gamma e_{j}(t-\tau )+u_{i}(t) \end{array}$$

**Figure 1:**
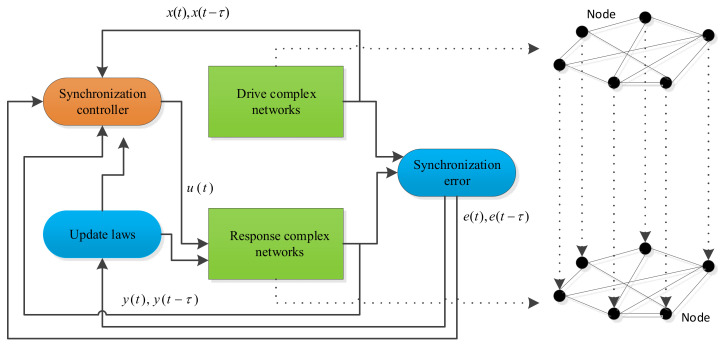
Block diagram of topology identification based on synchronization.

A block diagram of topology identification based on synchronization is shown in Figure 1. By designing a proper controller $u_{i}(t)$, the state of the response networks will be synchronized with the drive system based on Lyapunov stability theory.

**Remark** **1.** *According to the synchronization-based identification technique, adaptive controllers are designed to achieve synchronization between the response network and the drive network. This synchronization allows for the simultaneous estimation of topological parameters. That is to say, as time evolves,* $\lim_{t\to \infty }\left\|e_{i}(t)\right\|=0$ *for* i=1,2,…,N *and* $\lim_{t\to \infty }{\tilde{a}}_{ij}(t)=\lim_{t\to \infty }({\hat{a}}_{ij}(t)-a_{ij})=0$ *for* 1≤i,j≤N*. The unknown topology* ${\left(a_{ij}\right)}_{N\times N}$ *can be successfully identified by the matrix* ${\left({\hat{a}}_{ij}(t)\right)}_{N*N}$*. Additionally, it can overcome many of the mentioned shortcomings of other methods [8,9,10,11,12,13], except for requiring huge computation as well as a priori knowledge about the topological parameters. In addition, it does not require that the coupling topology matrix to be undirected [34,35].*

## 3. Main Results

In this section, a theories are presented to achieve topological identification for complex delayed networks with fixed and adaptive coupling strength.

### 3.1. Complex Delayed Networks with Fixed Coupling Strength

In this subsection, we design a synchronization-based topology (STO) observer for complex networks with delay and fixed coupling strength. A combination of a linear delayed feedback and an adaptive-feedback synchronization controller is designed.

**Theorem** **1.** *Suppose that Assumption 1 holds by using the following robust controller and update laws:*

(5) $$u_{i}(t)=-f(y_{i}(t))+f(x_{i}(t))-k_{i}(t)e_{i}(t)+\theta_{i}e_{i}(t-\tau )$$

(6) $${\dot{k}}_{i}(t)=\rho_{i}e_{i}^{T}(t)e_{i}(t)-\eta_{i}k_{i}(t)$$

(7) $${\dot{\hat{a}}}_{ij}(t)=-r_{ij}c(t)e_{i}^{T}(t)\Gamma y_{j}(t-\tau )$$

*where* $\theta_{i}>0,\rho_{i}>0,r_{ij}>0(i,j=1,2,\cdots ,N)$ *are arbitrary constants. The unknown topology can be estimated in terms of the variable errors between drive and response, where* $\rho_{i}>0,r_{ij}>0,\eta_{i}>0(i,j=1,2,\ldots ,N)$ *are arbitrary constants, and* $\theta_{i}$ *is a parameter that satisfies certain conditions. The coupling strength* c(t)=C*, and here,* C *is a positive constant. The unknown topology can be successfully estimated based on the principle of synchronization.*

**Proof.**  See Appendix A. □

**Remark** **2.** *In (1),* $a_{ij}$ *is the unknown parameter matrix, and* $a_{ij}$ *can be traced by* ${\hat{a}}_{ij}(t)$*. It is noteworthy that the unknown parameter matrix* $a_{ij}$ *is identified simultaneously when synchronization occurs. That is,* $e_{i}(t)\to 0$ *and* ${\tilde{a}}_{ij}(t)\to 0$ *as* t→∞*. Thus, when* t *is large enough,* ${\hat{a}}_{ij}(t)\to a_{ij}$*. This implies that the unknown weights* ${\hat{a}}_{ij}(t)$ *can be successfully estimated using the adaptive-feedback method proposed in Theorem 1.*

In terms of parameter identification issues based on synchronous control, the main focus is on designing corresponding control strategies and deriving synchronization stability conditions. Unlike the Kalman filter, the system’s synchronization error involves calculating the current response network nodes’ state values compared to the driving nodes. By designing suitable control methods, the aim is to gradually reduce the error to zero, thereby identifying the network’s topology structure.

### 3.2. Complex Delayed Networks with Adaptive Coupling Strength

In this subsection, we further investigate a TICDN with adaptive coupling strength.

**Theorem** **2.** *Suppose that Assumption 1 holds; the drive networks (1) and the response networks (2) can enable topology identification by using controller Equation (5) and update law Equations (6) and (7), and taking*

(8) $$\dot{\hat{\Theta }}(t)=-\psi \left(\left.\lambda_{2}\sum_{i=1}^{N}e_{i}^{T}(t)e_{i}(t)+\sum_{i=1}^{N}e_{i}^{T}(t-\tau )e_{i}(t-\tau )\right)\right.$$

*where* ψ>0 *is an arbitrary constant. If the trivial solution of the synchronization error (4) is asymptotically stable, then the matrix* A *can also be identified by update laws (7). The coupling strength* $c(t)=\hat{\Theta }(t)+\varepsilon$*,* ε*, and* $\hat{\Theta }(0)$ *are positive arbitrary constants. Meanwhile, as time reaches infinity, the coupling strength will finally converge to a constant, which is determined by the initial value* $\hat{\Theta }(0)$ *and the parameter* ε*.*

**Proof.**  See Appendix B. □

**Remark** **3.** *In fact, coupling strength is an essential factor in studying the dynamical behaviors of complex networks. In the case of adaptive coupling strength, the upper bound of* c(t) *does not need to be known beforehand from the inequality (8) of Theorem 2. As the time reaches infinity,* c(t) *will finally converge to a constant.*

**Remark** **4.** *The proposed controller (5) and adaptive control update laws (6) are used to ensure asymptotic synchronization between the drive and response networks. From Lyapunov’s function, it follows that the constants* $\rho_{i}>0$ *in Equation (6) ensure the convergence stability of Theorems 1 and 2 that the process of proof obtained. Obviously, the related results are applicable equally to the case of networks with known topology.*

**Remark** **5.** *In general, there are two ways to achieve synchronization in complex networks. One is by adjusting the self-adaptive coupling strength, which is reflected in the network by adjusting the connection weights between network nodes based on their dynamic relationships and states. This individual adjustment method is generally more difficult. The other way is to use an external controller. In this article, the presence of a synchronization controller significantly shortens the self-adaptive coupling strength adjustment process.*

## 4. Illustrative Examples

In this section, we give some examples and simulations to show the validity of the theoretical framework presented above. We consider a complex dynamical network composed of six identical nodes, with each node representing a Lorenz system. The mathematical description of the drive network can be further elaborated as follows:

$$\begin{array}{l} \left(\begin{array}{c} {\dot{x}}_{i1}(t)\\ {\dot{x}}_{i2}(t)\\ {\dot{x}}_{i3}(t) \end{array}\right)=\left(\begin{array}{c} 0\\ -x_{i1}(t)x_{i3}(t)-x_{i2}(t)\\ x_{i1}(t)x_{i2}(t) \end{array}\right)+\left(\begin{array}{ccc} x_{i2}(t)-x_{i1}(t) & 0 & 0\\ 0 & x_{i1}(t) & 0\\ 0 & 0 & -x_{i3}(t) \end{array}\right)\left(\begin{array}{c} a\\ b\\ c \end{array}\right)\\ \;\;\;\;+c(t)\sum_{j=1}^{6}a_{ij}\Gamma x_{j}(t-\tau ) \end{array}$$

where $x_{i}(t)={\left(x_{i1}(t),\;x_{i2}(t),\;x_{i3}(t)\right)}^{T}$ are the state variables, and the parameters vectors are as follows: ${\left(a,\;b,\;c\right)}^{T}={\left(10,\;28,\;8/3\right)}^{T}$. The outer coupling matrix $A=(a_{ij}{)}_{6\times 6}$ is chosen to be

$$A=\left[\begin{array}{cccccc} -2 & 2 & 0 & 0 & 0 & 0\\ 0 & -1 & 1 & 0 & 0 & 0\\ 0 & 0 & -3 & 0 & 0 & 3\\ 0 & 0 & 0 & -1 & 0 & 1\\ 0 & 2 & 0 & 0 & -2 & 0\\ 0 & 0 & 0 & 0 & 1 & -1 \end{array}\right]$$

The response networks can be described as

$$\begin{array}{l} \left(\begin{array}{c} {\dot{y}}_{i1}(t)\\ {\dot{y}}_{i2}(t)\\ {\dot{y}}_{i3}(t) \end{array}\right)=\left(\begin{array}{c} 0\\ -y_{i1}(t)y_{i3}(t)-y_{i2}(t)\\ y_{i1}(t)y_{i2}(t) \end{array}\right)+\left(\begin{array}{ccc} y_{i2}(t)-y_{i1}(t) & 0 & 0\\ 0 & y_{i1}(t) & 0\\ 0 & 0 & -y_{i3}(t) \end{array}\right)\left(\begin{array}{c} a\\ b\\ c \end{array}\right)\\ \;\;\;\;+c(t)\sum_{j=1}^{6}{\hat{a}}_{ij}\Gamma y_{j}(t-\tau )+u_{i}(t) \end{array}$$

where $y_{i}(t)={\left(\begin{array}{ccc} y_{i1}(t), & y_{i2}(t), & y_{i3}(t) \end{array}\right)}^{T}$ are the response state vectors, $u_{i}(t)$ represents the controllers to be designed, the time delay τ=0.5, and the network inner-coupling matrix is $\Gamma =I_{6\times 6}$.

The chosen coefficients in Equations (5)–(7) are as follows: $\theta_{i}=0.01$, $\rho_{i}=0.1$$,\gamma_{ij}=0.5$, i,j=1,2,⋯,6.

In the numerical simulations, the initial values are given as follows:

$$x_{i}(0)={\left[1+0.8i,1+0.7i,0.8i\right]}^{T};\;y_{i}(0)={\left[-1+8i,-1+i,-8i\right]}^{T},\;i=1,2,\cdots ,6;$$

$${\hat{a}}_{ij}(0)=1\;,k_{i}(0)=1+i\;,i,j=1,2,\cdots ,6.$$

### 4.1. Identification Topology of Complex Networks with Fixed Coupling Strength

#### 4.1.1. The Effectiveness of the Proposed STO

**Figure 2:**
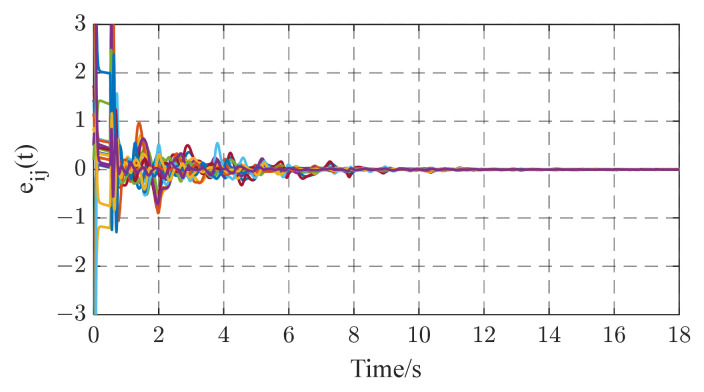
The temporal evolution of the synchronization error $e_{ij}(i=1,2,\cdots ,6;j=1,2,3)$.

**Figure 3:**
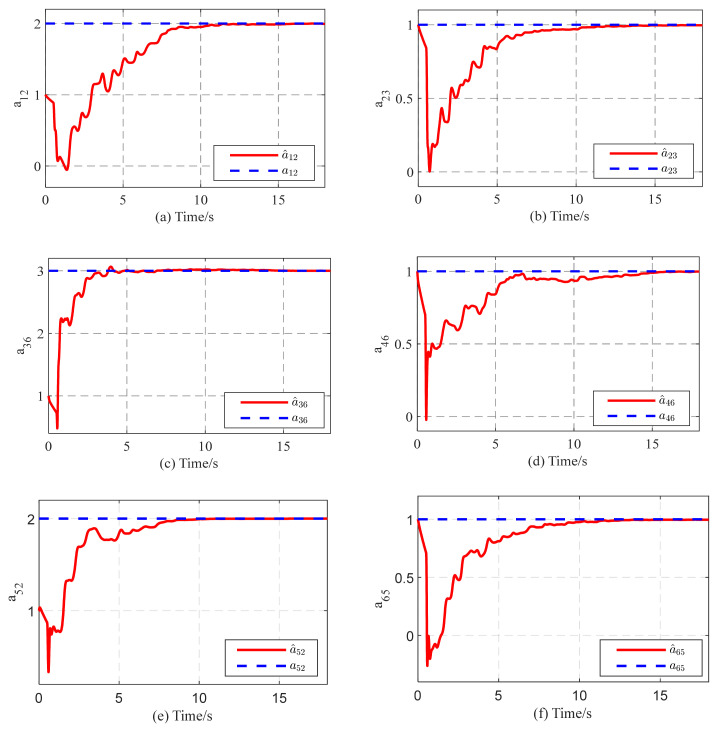
Partial identification results (**a**–**f**) of network topology with the fixed coupling strength are displayed.

**Figure 4:**
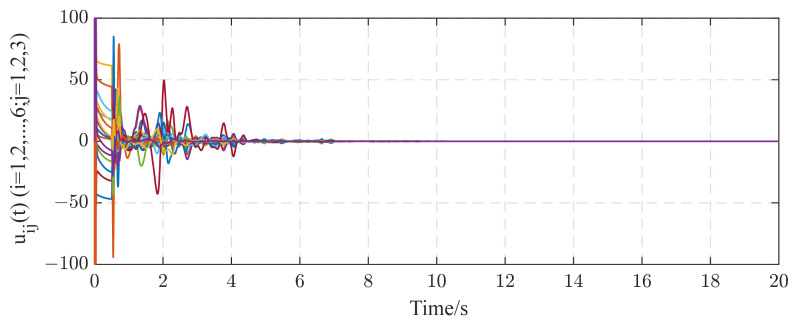
The control inputs $u_{ij}(t)(i=1,2,\ldots ,6;j=1,2,3)$ under the networks with fixed coupling strength.

In this subsection, we consider a TICDN with fixed coupling strength. Without loss of generality, we assume that the coupling strength is c=1. Figure 2 and Figure 3 display the numerical results based on Equations (5)–(7). Figure 1 shows the temporal evolution of the synchronization errors (4). As time progresses, the synchronization errors gradually approach zero, indicating successful synchronization. Figure 2 displays the estimation of the six existing edges, $a_{12}$, $a_{23}$, $a_{36}$, $a_{46}$, $a_{52}$, $a_{65}$. These figures indicate that the topological parameter ${\hat{a}}_{ij}$ can converge to the real valve of $a_{ij}\;(i,j=1,2\cdots ,6)$ by using the proposed STO. Figure 4 illustrates the temporal evolution of the effective control inputs $u_{i}(t)=[u_{i1}(t);u_{i2}(t);u_{i3}(t)]\;(i=1,2,\ldots ,6)$ via Theorem 1.

#### 4.1.2. Comparison with Other Methods

In this part, the identification error of the proposed STO is also compared with two classical methods, including the observer-based adaptive pinning control (APC) in [22] and the observer-based linear feedback control (LFC) in [25]. The comparison methods have the same initial condition.

(i) Performance indices

To facilitate quantitative comparison and analysis, the following three performance indices are given. Here, T represents simulation time. The identification error is denoted as E(t).

1. Minimum identification time (MIT): the time needed for successful identification when E(t) decreases to a specific value ω. In this study, we adopt a ω value of $3\times 10^{-3}$.
2. The integral of the identification error (IE) is calculated as follows:
  $$IE=\int_{0}^{T}E(t)dt$$
  where
  $$E(t)=\sum_{i=1}^{N}\sum_{j=1}^{N}\left|{\hat{a}}_{ij}-a_{ij}\right|/N^{2}$$
3. The final error result of the identification topology is given by the steady-state error (SE):
  $$SE=\lim_{t\to T}E(t)$$

(ii) Identification results

**Figure 5:**
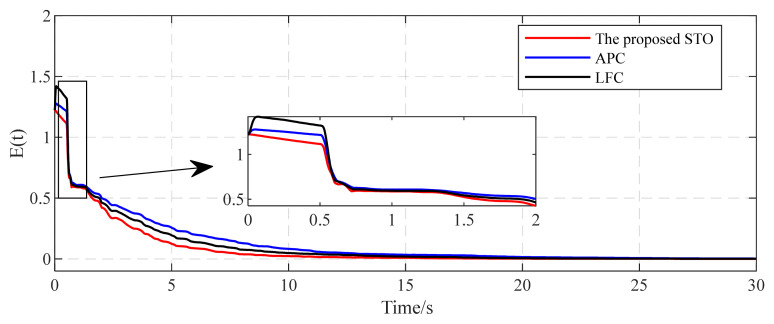
Comparison of the identification error E(t) using different methods.

Figure 5 shows the convergence process of identification errors using different methods. The relative performance indices in terms of IE, SE, and MIT in Table 1 are calculated based on the curves shown in Figure 5. As shown in Table 1, compared with the APC [22] and LFC [25] methods, the proposed STO has decreased performance index IE by 34.32% and 18.73%, respectively. In addition, the proposed STO achieves the smallest steady-state identification error SE among the three different methods. These results provide evidence that the proposed STO has better identification performance and higher accuracy than the others. Additionally, upon comparing the minimum identification times, the convergence speed of the proposed STO is obviously faster than others. The identification error is less than $3\times 10^{-3}$, achieved with the shortest time of 18.33 s.

Considering the aforementioned discussion, it can be concluded that the proposed STO method outperforms the other two methods in terms of its smaller tracking error, faster convergence rate, and higher identification precision.

### 4.2. Identification Topology of Complex Networks with Adaptive Coupling Strength

In the last section, we discuss the effect of the proposed STO in solving a TICDN with a fixed coupling strength. However, in the real world, the coupling strength in complex networks may not be static, but instead, an adaptive parameter. The coupling strength is dynamically adjusted based on changes in the environment or the network itself (e.g., neural networks, wireless sensor networks, and social networks). In this section, we discuss the fact that the coupling strength of complex networks is adaptive during the identification process. The remaining initial arguments are the same as in Section 4.1. We take the initial value of $\hat{\Theta }(0)=2,\varepsilon =0.01$.

**Figure 6:**
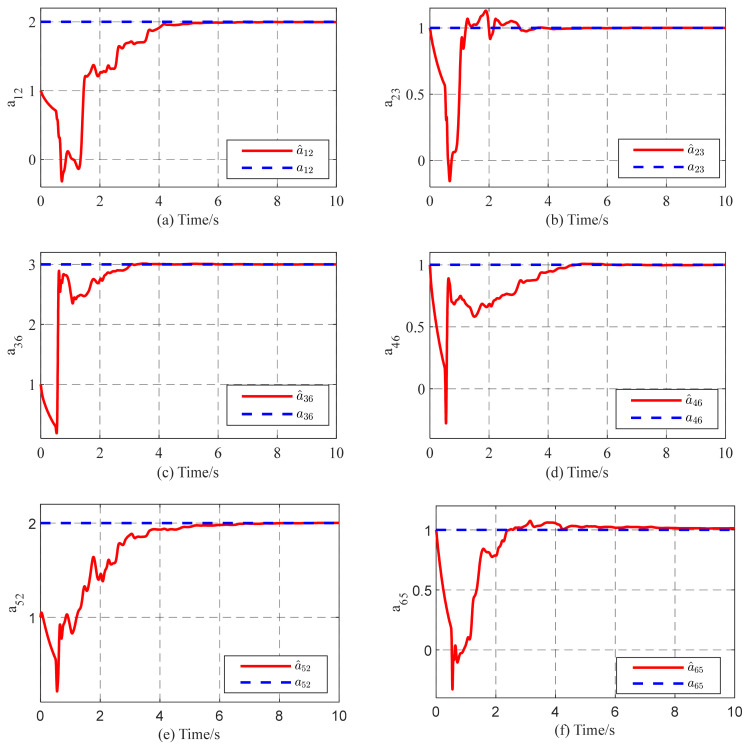
Partial identification results (**a**–**f**) of network topology with adaptive coupling strength.

**Figure 7:**
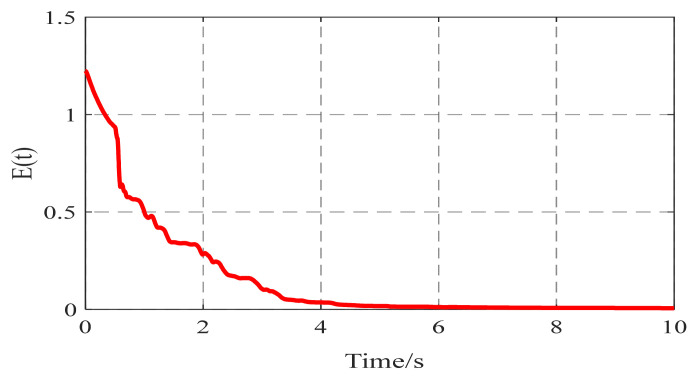
The temporal evolution of the topology identification error E(t).

**Figure 8:**
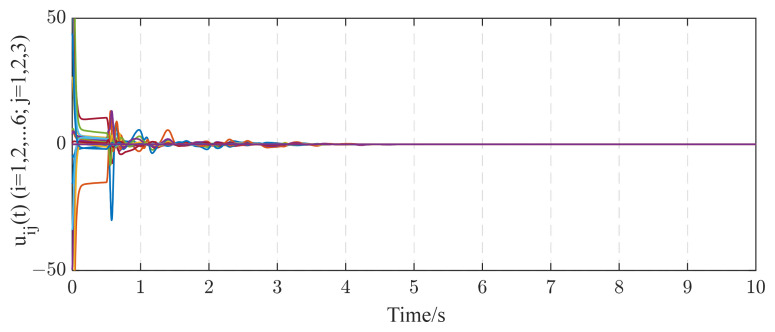
The control inputs $u_{ij}(t)(i=1,2,\ldots ,6;j=1,2,3)$ under the networks with adaptive coupling strength.

Figure 6 shows the identification results of the six existing edge elements in the coupling matrix $A_{6*6}$, which converge to their real values as time increase. According to Figure 6, the identification error E(t) tends to zero. Figure 6 and Figure 7 indicate that the unknown topology of complex networks are determined successfully by using controller (5) and update laws (6)–(8). Figure 8 shows the temporal evolution of the effective control inputs $u_{i}(t)=[u_{i1}(t);u_{i2}(t);u_{i3}(t)](i=1,2,\ldots ,6)$.

**Figure 9:**
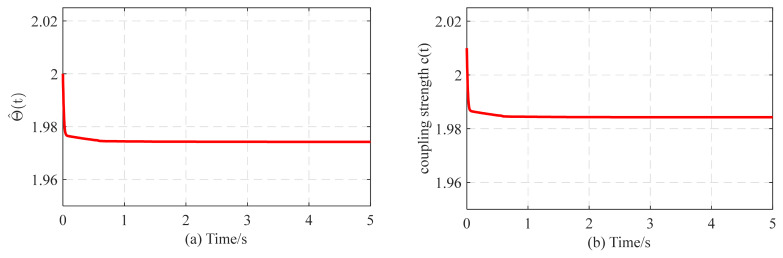
The temporal evolution of (**a**) $\hat{\Theta }(t)$ and (**b**) adaptive coupling strength c(t).

Figure 9a shows the variation in $\hat{\Theta }(t)$ according to the adaptive law (15). Meanwhile, the coupling strength c(t) adjusts itself to reach a certain constant value, as depicted in Figure 9b. In Figure 9, the topology of complex networks with adaptive coupling strength can be identified correctly and effectively, which aligns with the findings presented in Theorem 2.

### 4.3. Robustness Test for Topology Identification

#### 4.3.1. Complex Networks with Fixed Coupling Strength

Case 1: robustness to parameter variation

**Figure 10:**
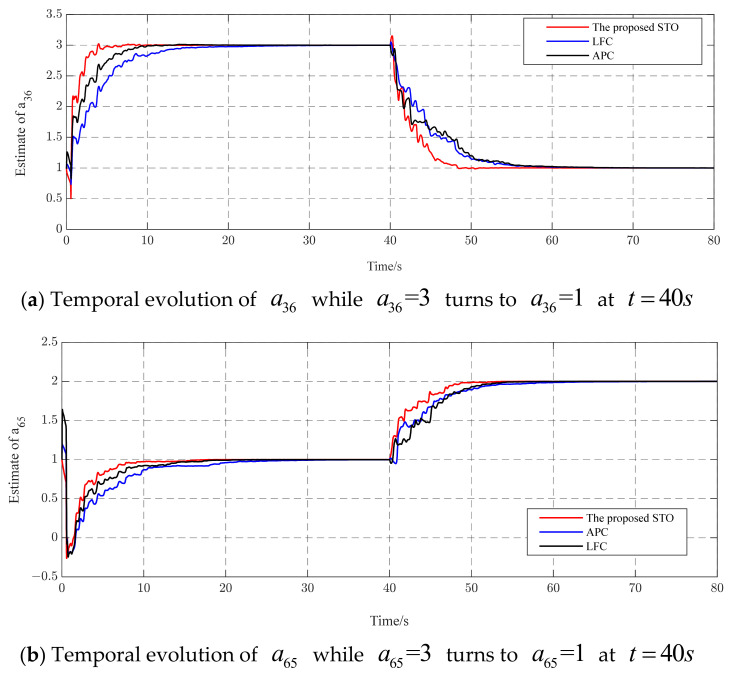
Identification results of (**a**) ${\hat{a}}_{36}$ and (**b**) ${\hat{a}}_{65}$.

Generally speaking, it is necessary to consider robustness when designing an observer. In the process of parameter identification, one of the more common concerns is the problem of the robustness of the proposed STO, which is influenced by the variation in unknown topological parameters. In the real world, the connections between nodes may strengthen or weaken over time. Here, we assumed that a portion of the unknown topological parameter $a_{ij}$ is affected at a given time. Figure 10 shows the temporal evolution of ${\hat{a}}_{36},{\hat{a}}_{65}$ when $a_{36}=3,a_{65}=1$ turns to $a_{36}=1,a_{65}=2$ at t=40 s. It is evident that all of the methods can automatically and accurately track over time. However, the proposed STO exhibits a noticeably faster convergence speed compared to the other methods. This indicates that the proposed STO possesses rapid tracking capabilities and strong robustness in the face of variations in topological parameters.

Case 2: robustness to external disturbances

In order to prove the reliability of the proposed STO scheme, a robustness test is conducted on complex networks with external disturbances in this sub-part. Here, the external disturbances chosen are s(t)=sin(t)(28≤t≤30). The coupling matrix Γ is replaced with $\bar{\Gamma }={\left[\Gamma s(t)\right]}_{6\times 6}$.

**Figure 11:**
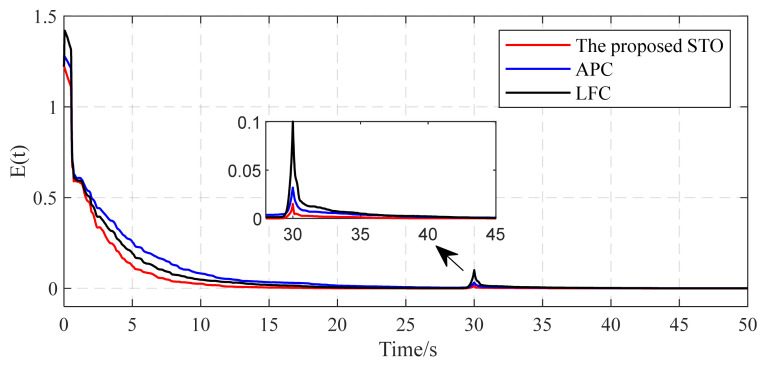
Comparison of identification error E(t) with external disturbances.

Figure 11 shows topology identification error E(t) curves under different methods. It is seen that the proposed STO exhibits stronger anti-disturbance capability. The estimations of all unknown topological parameters converge to their actual values within seconds. The performance indices in Table 2 are calculated using the curves depicted in Figure 10. Upon comparing the identification performance indices, it is clear that the proposed STO shows its superiority across all performance indices when compared to the other two methods. From these simulation results, we can see that the proposed STO has better robustness capability, including a lower identification error and shorter convergence time, particularly in the presence of external disturbances affecting the complex networks.

#### 4.3.2. Complex Networks with Adaptive Coupling Strength

**Figure 12:**
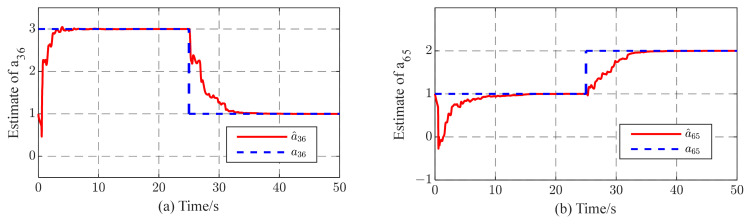
Tracking results while $a_{36}=3,a_{65}=1$ turns to $a_{36}=1,a_{65}=2$ at t= s; (**a**,**b**) temporal evolution of ${\hat{a}}_{36},{\hat{a}}_{65}$, respectively.

**Figure 13:**
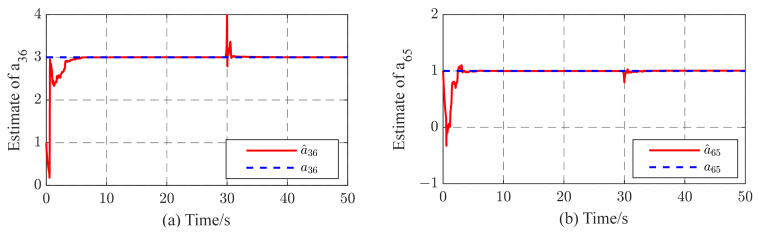
(**a**,**b**) Temporal evolution of estimates ${\hat{a}}_{36},{\hat{a}}_{65}$, respectively, in complex dynamic network with external disturbances at t= s.

To illustrate the robustness of the identification method to cases where the coupling strength is unknown, in our simulations, we also consider the uncertainties and external perturbations of complex networks. Figure 12 and Figure 13 show the simulation plots. From these simulations, it can be observed that the proposed method exhibits not only excellent tracking performance but also remarkable robustness against parametric variations and external disturbances. As illustrated in Figure 12, the proposed method demonstrates an ability to rapidly track changes in the unknown parameter while maintaining consistently good performance. In addition, as noted in Figure 13, the topological parameters ${\hat{a}}_{36}$ and ${\hat{a}}_{65}$ can recover the original defined values even after experiencing external disturbances. In addition, after 5~7 s, the state $y_{i}$(i=1,2…,6) quickly synchronizes with the state $x_{i}$.

## 5. Conclusions

In this paper, a novel synchronization-based topology observer for TICDNs is presented. Two kinds of network models are considered: online topology estimation of complex networks (i) with coupling delay and fixed coupling strength and (ii) with coupling delay and adaptive coupling strength. Based on the stability theorem approach, several useful estimation criteria for the real-time identification of topology weights are obtained. The identification method proposed is not only applied to complex delayed networks with fixed coupling strength, but is also suitable for complex delayed networks with adaptive coupling strength during the identification process. Simulation results are obtained to validate the effectiveness of the proposed STO using Theorems 1 and 2. The synchronization errors of the networks with fixed or adaptive coupling strength all tend to zero as time evolves in Figure 2 and Figure 7. The unknown topology can be successfully identified within seconds, as shown in Figure 3 and Figure 6. In other words, achieving synchronization ensures the correct identification of topological structures. Even though cases of external perturbations and parameter variation exist in complex networks, as shown in Figure 10 and Figure 13, the topological parameter identification results are still correct. In addition, under the same simulation time and the same identification error requirement, based on the computational indicators in Table 1 and Table 2, the proposed STO achieves high accuracy and stability. In addition, from an application point of view, the proposed method is capable of the automatic online adjustment of parameters, which may be easily implemented in practical applications. However, it would also be very interesting to study time-varying delays between the nodes, and we will further investigate this problem in the future.

## Figures and Tables

**Table 1:** Comparison of the performance indices with the different methods.

Method Indices	IE	SE	MIT(s)
The proposed STO	2.5540 (65.77%) *	5.6230 × 10^−5^ (2.129%)	18.3301 (61.96%)
LFC [24]	3.8829 (100.0%)	2.6411 × 10^−3^ (100.0%)	29.5825 (100.0%)
APC [35]	3.2815 (84.50%)	1.1611 × 10^−3^ (43.96%)	22.8965 (77.39%)

* (ζ) is the relative ratio. The chosen maximum value at each index is denoted as Δ, with the definition of $\zeta =\frac{\Omega }{\Delta }\times 100\%$ provided, where Ω signifies the remaining value at each index.

**Table 2:** Comparison of performance indices with different methods.

Method Indices	IE	SE	MIT(s)
The proposed STO	2.5776 (65.32%)	4.1985 × 10^−6^ (1.247%)	30.4419 (82.07%)
LFC [24]	3.9458 (100.0%)	3.3647 × 10^−4^ (100.0%)	37.0951 (100.0%)
ASC [35]	3.3868 (85.97%)	1.8896 × 10^−4^ (56.16%)	36.7364 (99.03%)

## Data Availability

Data are contained within the article.

## Appendix A

**Proof.** Consider the following Lyapunov function

$$\begin{array}{ll} V(t) & =\frac{1}{2}\sum_{i=1}^{N}e_{i}^{T}(t)e_{i}(t)+\frac{1}{2}\sum_{i=1}^{N}\sum_{j=1}^{N}\frac{1}{r_{ij}}{\left({\hat{a}}_{ij}-a_{ij}\right)}^{2}+\frac{1}{2}\sum_{i=1}^{N}{\left(k_{i}(t)\right)}^{2}\;\\ & +\frac{NC}{2}\sum_{i=1}^{N}\int_{t-\tau }^{t}{\left(\Gamma e_{i}(\mu )\right)}^{T}(\Gamma e_{i}(\mu ))d\mu +\frac{1}{2}\sum_{i=1}^{N}\int_{t-\tau }^{t}\theta_{i}{\left(e_{i}(\mu )\right)}^{T}(e_{i}(\mu ))d\mu \end{array}$$

where $\theta_{i}^{*}$ is a sufficient constant to be determined. Substituting Equation (4) into the time derivative of V(t), it can be expressed as

$$\begin{array}{cc} \dot{V}\left(t\right) & =\sum_{i=1}^{N}e_{i}^{T}\left\{f(y_{i})\right.-f\left(x_{i}\right)+C\sum_{j=1}^{N}({\hat{a}}_{ij}-a_{ij})\Gamma y_{j}\left(t-\tau \left(t\right)\right)+C\sum_{j=1}^{N}a_{ij}\Gamma e_{j}\left(t-\tau \right)+\left.u_{i}\left(t\right)\right\}\\ & \;+\sum_{i=1}^{N}\sum_{j=1}^{N}\frac{1}{r_{ij}}\left({\hat{a}}_{ij}-a_{ij}\right){\dot{\hat{a}}}_{ij}+\sum_{i=1}^{N}k_{i}\left(t\right){\dot{k}}_{i}^{T}\left(t\right)+\frac{NC}{2}\sum_{i=1}^{N}{\left(\Gamma e_{i}\left(t\right)\right)}^{T}\left(\Gamma e_{i}\left(t\right)\right)\\ & \;-\frac{NC}{2}\sum_{i=1}^{N}{\left(\Gamma e_{i}\left(t-\tau \right)\right)}^{T}\left(\Gamma e_{i}\left(t-\tau \right)\right)+\frac{\theta_{i}}{2}\sum_{i=1}^{N}e_{i}{\left(t\right)}^{T}e_{i}\left(t\right)-\frac{\theta_{i}}{2}\sum_{i=1}^{N}e_{i}{\left(t-\tau \right)}^{T}e_{i}\left(t-\tau \right) \end{array}$$

According to Equations (5)–(7), this implies that

$$\begin{array}{cc} \dot{V}\left(t\right) & =\sum_{i=1}^{N}e_{i}^{T}\left(t\right)C\sum_{j=1}^{N}({\hat{a}}_{ij}-a_{ij})\Gamma y_{j}\left(t-\tau \left(t\right)\right)+C\sum_{j=1}^{N}a_{ij}\Gamma e_{j}\left(t-\tau \right)-k_{i}\left(t\right)e_{i}\left(t\right)+\theta_{i}\left.e_{i}\left(t-\tau \right)\right\}\\ & \;-\sum_{i=1}^{N}\sum_{j=1}^{N}\frac{1}{r_{ij}}\left({\hat{a}}_{ij}-a_{ij}\right)r_{ij}c\left(t\right)e_{i}^{T}\left(t\right)\Gamma y_{j}\left(t-\tau \left(t\right)\right)+\sum_{i=1}^{N}\frac{1}{\rho_{i}}k_{i}\left(t\right)\left(\rho_{i}e_{i}^{T}\left(t\right)e_{i}\left(t\right)-\eta_{i}k_{i}\left(t\right)\right)\\ & \;+\frac{NC}{2}\sum_{i=1}^{N}{\left(\Gamma e_{i}\left(t\right)\right)}^{T}\left(\Gamma e_{i}\left(t\right)\right)-\frac{NC}{2}\sum_{i=1}^{N}{\left(\Gamma e_{i}\left(t-\tau \right)\right)}^{T}\left(\Gamma e_{i}\left(t-\tau \right)\right)\\ & \;+\frac{\theta_{i}}{2}\sum_{i=1}^{N}e_{i}{\left(t\right)}^{T}e_{i}\left(t\right)-\frac{\theta_{i}}{2}\sum_{i=1}^{N}e_{i}{\left(t-\tau \right)}^{T}e_{i}\left(t-\tau \right) \end{array}$$

Then, we can obtain

$$\begin{array}{ll} \dot{V}(t) & =\theta_{i}\sum_{i=1}^{N}e_{i}^{T}(t)e_{i}(t-\tau )+C\sum_{i=1}^{N}\sum_{j=1}^{N}a_{ij}e_{i}^{T}(t)\Gamma e_{j}(t-\tau )-\sum_{i=1}^{N}\frac{\eta_{i}}{\rho_{i}}k_{i}^{2}(t)\;\\ & +\frac{NC}{2}\sum_{i=1}^{N}{\left(\Gamma e_{i}(t)\right)}^{T}(\Gamma e_{i}(t))-\frac{NC}{2}\sum_{i=1}^{N}{\left(\Gamma e_{i}(t-\tau )\right)}^{T}(\Gamma e_{i}(t-\tau ))\\ & +\frac{\theta_{i}}{2}\sum_{i=1}^{N}e_{i}{\left(t\right)}^{T}e_{i}(t)-\frac{\theta_{i}}{2}\sum_{i=1}^{N}e_{i}{\left(t-\tau \right)}^{T}e_{i}(t-\tau ) \end{array}$$

In accordance with Lemma 1, the following can be obtained:

$$\begin{array}{ll} \dot{V}(t) & \leq \theta_{i}\sum_{i=1}^{N}e_{i}^{T}(t)e_{i}(t-\tau )+\frac{1}{2}C\sum_{i=1}^{N}\sum_{j=1}^{N}a_{ij}^{2}e_{i}^{T}(t)e_{i}(t)\\ & +\frac{1}{2}C\sum_{i=1}^{N}\sum_{j=1}^{N}{\left(\Gamma e_{i}(t-\tau )\right)}^{T}(\Gamma e_{i}(t-\tau ))+\frac{CN}{2}\sum_{i=1}^{N}{\left(\Gamma e_{i}(t)\right)}^{T}(\Gamma e_{i}(t))\\ & -\frac{NC}{2}\sum_{i=1}^{N}{\left(\Gamma e_{i}(t-\tau )\right)}^{T}(\Gamma e_{i}(t-\tau ))+\frac{\theta_{i}}{2}\sum_{i=1}^{N}e_{i}{\left(t\right)}^{T}e_{i}(t)-\frac{\theta_{i}}{2}\sum_{i=1}^{N}e_{i}{\left(t-\tau \right)}^{T}e_{i}(t-\tau ) \end{array}$$

Assume that $e(t)={\left[e_{1}^{T}(t),e_{2}^{T}(t),\cdots ,e_{N}^{T}(t)\right]}^{T}$, and suppose that Lemma 1 and Assumption 1 hold; it can be further derived as

$$\begin{array}{ll} \dot{V}(t) & \leq \frac{1}{2}\theta_{i}e^{T}(t)e(t)+\frac{1}{2}\theta_{i}e^{T}(t-\tau )e(t-\tau )+\frac{1}{2}Cl^{2}Ne^{T}(t)e(t)\\ & +\frac{1}{2}CN(\Gamma e{\left(t-\tau \right)}^{T}(\Gamma e(t-\tau ))+\frac{CN}{2}\lambda_{\Gamma^{T}\Gamma }e^{T}(t)e(t)\\ & -\frac{CN}{2}(\Gamma e{\left(t-\tau \right)}^{T}(\Gamma e(t-\tau ))+\frac{\theta_{i}}{2}e^{T}(t)e(t)-\frac{\theta_{i}}{2}e^{T}(t-\tau )e(t-\tau )\\ & =(\theta_{i}+\frac{1}{2}Cl^{2}N+\frac{CN}{2}\lambda_{1})e^{T}(t)e(t) \end{array}$$

Let $\theta^{*}=\underset{1\leq i\leq N}{\max}\left\{\theta_{i}\right\}$$\lambda_{1}$ be the maximum eigenvalue of the matrix $\Gamma^{T}\Gamma$. Choose an appropriate parameter $\theta^{*}$ that meets the following conditions:

$$\frac{1}{2}Cl^{2}N+\theta^{*}+\frac{CN}{2}\lambda_{1}<0$$

We have $\dot{V}\leq -e^{T}(t)e(t)$. According to the Lyapunov stability theory, the synchronization error (4) can be asymptotically stable. This completes the proof. □

## Appendix B

**Proof.** Consider the following Lyapunov function:

$$\begin{array}{ll} V(t) & =\frac{1}{2}\sum_{i=1}^{N}e_{i}^{T}(t)e_{i}(t)+\frac{1}{2}\sum_{i=1}^{N}\sum_{j=1}^{N}\frac{1}{r_{ij}}{\left({\hat{a}}_{ij}-a_{ij}\right)}^{2}+\frac{1}{2}\sum_{i=1}^{N}{\left(k_{i}(t)\right)}^{2}+\frac{1}{4\psi }{\hat{\Theta }}^{2}(t)\\ & +\frac{N\varepsilon }{2}\sum_{i=1}^{N}\int_{t-\tau }^{t}{\left(\Gamma e_{i}(\mu )\right)}^{T}(\Gamma e_{i}(\mu ))d\mu +\frac{1}{2}\sum_{i=1}^{N}\int_{t-\tau }^{t}\theta_{i}e_{i}^{T}(\mu )e_{i}(\mu )d\mu \end{array}$$

Upon differentiating V(t), and then substituting Equations (4)–(7), one obtains

$$\begin{array}{cc} \dot{V}\left(t\right) & \leq \left(\hat{\Theta }+\varepsilon \right)\sum_{i=1}^{N}\sum_{j=1}^{N}a_{ij}e_{i}^{T}\left(t\right)\Gamma e_{j}\left(t-\tau \right)+\frac{1}{2}\sum_{i=1}^{N}\theta_{i}e_{i}^{T}\left(t\right)e_{i}\left(t\right)+\frac{1}{2}\sum_{i=1}^{N}\theta_{i}e_{i}{\left(t-\tau \right)}^{T}e_{i}\left(t-\tau \right)\;\\ & \;+\frac{N\varepsilon }{2}\lambda_{1}\sum_{i=1}^{N}e_{i}^{T}\left(t\right)e_{i}\left(t\right)-\frac{N\varepsilon }{2}\sum_{i=1}^{N}{\left(\Gamma e_{i}\left(t-\tau \right)\right)}^{T}\left(\Gamma e_{i}\left(t-\tau \right)\right)-\sum_{i=1}^{N}\frac{\eta_{i}}{\rho_{i}}k_{i}^{2}\left(t\right)\;\\ & \;+\frac{1}{2}\sum_{i=1}^{N}\theta_{i}e_{i}{\left(t\right)}^{T}e_{i}\left(t\right)-\frac{1}{2}\sum_{i=1}^{N}\theta_{i}e_{i}{\left(t-\tau \right)}^{T}e_{i}\left(t-\tau \right)+\frac{1}{2\psi }\hat{\Theta }\left(t\right)\dot{\hat{\Theta }}\left(t\right)\; \end{array}$$

If Lemma 1 holds, we have

$$\begin{array}{ll} \dot{V}(t) & \leq \frac{1}{2}\varepsilon l^{2}N\sum_{i=1}^{N}e_{i}^{T}(t)e_{i}(t)+\frac{1}{2}\varepsilon N\sum_{j=1}^{N}{\left(\Gamma e_{j}(t-\tau )\right)}^{T}(\Gamma e_{j}(t-\tau ))\\ & +\hat{\Theta }(t)\left(\sum_{i=1}^{N}\sum_{j=1}^{N}a_{ij}e_{i}^{T}(t)\Gamma e_{j}(t-\tau )\right)+\sum_{i=1}^{N}\theta_{i}e_{i}{\left(t\right)}^{T}e_{i}(t)+\frac{N\varepsilon }{2}\lambda_{1}\sum_{i=1}^{N}e_{i}^{T}(t)e_{i}(t)\\ & -\frac{N\varepsilon }{2}\sum_{i=1}^{N}{\left(\Gamma e_{i}(t-\tau )\right)}^{T}(\Gamma e_{i}(t-\tau ))+\frac{1}{2\psi }\hat{\Theta }(t)\dot{\hat{\Theta }}(t) \end{array}$$

Then, we can further obtain

$$\begin{array}{ll} \dot{V}(t) & \leq \frac{1}{2}\varepsilon l^{2}N\sum_{i=1}^{N}e_{i}^{T}(t)e_{i}(t)+\hat{\Theta }(t)\left(\sum_{i=1}^{N}\sum_{j=1}^{N}a_{ij}e_{i}^{T}(t)\Gamma e_{j}(t-\tau )\right)+\sum_{i=1}^{N}\theta_{i}e_{i}{\left(t\right)}^{T}e_{i}(t)\\ & +\frac{N\varepsilon }{2}\lambda_{1}\sum_{i=1}^{N}e_{i}^{T}(t)e_{i}(t)+\frac{1}{2\psi }\hat{\Theta }(t)\dot{\hat{\Theta }}(t) \end{array}$$

Assume that $e(t)={\left[e_{1}^{T}(t),e_{2}^{T}(t),\cdots ,e_{N}^{T}(t)\right]}^{T}$; by using the Kronecker product, we obtain

$$\dot{V}(t)=(\frac{1}{2}\varepsilon l^{2}N+\theta_{i}+\frac{N\varepsilon }{2}\lambda_{1})e^{T}(t)e(t)+\hat{\Theta }(t)e^{T}(t)(A⊗\Gamma )e(t-\tau )+\frac{1}{2\psi }\hat{\Theta }(t)\dot{\hat{\Theta }}(t)$$

According to Lemma 1, one obtains

$$\begin{array}{ll} \dot{V}(t) & \leq (\frac{1}{2}\varepsilon l^{2}N+\theta_{i}+\frac{N\varepsilon }{2}\lambda_{1})e^{T}(t)e(t)+\frac{1}{2}\hat{\Theta }(t)e^{T}(t){\left(A⊗\Gamma \right)}^{T}(A⊗\Gamma )e(t)\\ & +\frac{1}{2}\hat{\Theta }(t)e^{T}(t-\tau )e(t-\tau )-\frac{1}{2}\hat{\Theta }(t)(\lambda_{1}e^{T}(t)e(t)+e^{T}(t-\tau )e(t-\tau )) \end{array}$$

Let $\lambda_{2}=\lambda_{\max(A⊗\Gamma )}$; we have

$$\begin{array}{ll} \dot{V}(t) & \leq (\frac{1}{2}\varepsilon l^{2}N+\theta_{i}+\frac{N\varepsilon }{2}\lambda_{1})e^{T}(t)e(t)+\frac{1}{2}\lambda_{2}\hat{\Theta }(t)e^{T}(t)e(t)\\ & +\frac{1}{2}\hat{\Theta }(t)e^{T}(t-\tau )e(t-\tau )-\frac{1}{2}\hat{\Theta }(t)(\lambda_{2}e^{T}(t)e(t)+e^{T}(t-\tau )e(t-\tau ))\\ & =(\frac{1}{2}\varepsilon l^{2}N+\theta_{i}+\frac{N\varepsilon }{2}\lambda_{1})e^{T}(t)e(t) \end{array}$$

Choose an appropriate parameter $\theta_{i}$ that meets the following conditions:

$$\frac{1}{2}\varepsilon l^{2}N+\theta_{i}+\frac{N\varepsilon }{2}\lambda_{1}<0\;i=1,2,\ldots N$$

We have $\dot{V}\leq -e^{T}(t)e(t)$. This completes the proof. From Theorem 2, with update law (8), since $\dot{\hat{\Theta }}(t)\to 0$, $\hat{\Theta }(t)$ will converge to a constant, and $c(t)\to c_{0}+\varepsilon$ with t→∞, where $c_{0}$ is a positive constant. □
